# Improved synthesis of 6-bromo-7-[^11^C]methylpurine for clinical use

**DOI:** 10.1186/s41181-024-00240-8

**Published:** 2024-02-09

**Authors:** Toshimitsu Okamura, Tatsuya Kikuchi, Masanao Ogawa, Ming-Rong Zhang

**Affiliations:** 1 Department of Advanced Nuclear Medicine Sciences, Institute for Quantum Medical Science, National Institutes for Quantum Science and Technology, 4-9-1 Anagawa, Inage-ku, Chiba, 263-8555 Japan; 2 SHI Accelerator Service, Ltd., 7-1-1 Nishigotanda, Shinagawa-ku, Tokyo, 141-0031 Japan

**Keywords:** 6-Bromopurine, Carbon-11, Methylation, MRP1, Regioselectivity

## Abstract

**Background:**

Multidrug resistance-associated protein 1 (MRP1), an energy-dependent efflux pump, is expressed widely in various tissues and contributes to many physiological and pathophysiological processes. 6-Bromo-7-[^11^C]methylpurine ([^11^C]7m6BP) is expected to be useful for the assessment of MRP1 activity in the human brain and lungs. However, the radiochemical yield (RCY) in the synthesis of [^11^C]7m6BP was low, limiting its clinical application, because the methylation of the precursor with [^11^C]CH_3_I provided primarily the undesired isomer, 6-bromo-9-[^11^C]methylpurine ([^11^C]9m6BP). To increase the RCY of [^11^C]7m6BP, we investigated conditions for improving the [^11^C]7m6BP/[^11^C]9m6BP selectivity of the methylation reaction.

**Results:**

[^11^C]7m6BP was manually synthesized via the methylation of 6-bromopurine with [^11^C]CH_3_I in various solvents and at different temperatures in the presence of potassium carbonate for 5 min. Several less polar solvents, including tetrahydrofuran (THF), 2-methyltetrahydrofuran (2-MeTHF), and ethyl acetate (AcOEt) improved the [^11^C]7m6BP/[^11^C]9m6BP selectivity from 1:1 to 2:1, compared with the conventionally used solvents for the alkylation of 6-halopurines, acetone, acetonitrile, and *N*,*N*-dimethylformamide. However, a higher temperature (140 °C or 180 °C) was needed to progress the ^11^C-methylation in the less polar solvents, and the manual conditions could not be directly translated to an automated synthesis. [^11^C]Methyl triflate ([^11^C]CH_3_OTf) was thus used as a methylating agent to increase the conversion at a lower temperature. The ^11^C-methylation using [^11^C]CH_3_OTf at 100 °C proceeded efficiently in THF, 2-MeTHF, and AcOEt with maintenance of the improved selectivity. Starting from 28 to 34 GBq [^11^C]CO_2_, [^11^C]7m6BP was produced with 2.3–2.6 GBq for THF, 2.7–3.3 GBq for AcOEt, and 2.8–3.9 GBq for 2-MeTHF at approximately 30 min after the end of bombardment (*n* = 3 per solvent). The isolated RCYs (decay corrected) for THF, 2-MeTHF, and AcOEt were 24–28%, 29–35%, and 22–31% (*n* = 3), respectively.

**Conclusions:**

The use of THF, 2-MeTHF, and AcOEt improved the [^11^C]7m6BP/[^11^C]9m6BP selectivity in the methylation reaction, and the improved method provided [^11^C]7m6BP with sufficient radioactivity for clinical use.

**Supplementary Information:**

The online version contains supplementary material available at 10.1186/s41181-024-00240-8.

## Background

Multidrug resistance-associated protein 1 (MRP1) is a member of the adenosine triphosphate (ATP)-binding cassette superfamily of transporters and pumps various compounds, including therapeutic agents and physiological substances, out of cells using the energy of ATP hydrolysis (Bakos and Homolya 2007; Cole et al. 1992). This protein is widely expressed in normal tissues, including the brain, lung, heart, and kidney (Bakos and Homolya 2007; Flens et al. 1996). In addition to conferring multidrug resistance (Leslie et al. 2005; Löscher and Potschka 2005), changes in MRP1 activity or expression are associated with brain and lung diseases (Krohn et al. 2011, 2015; Qosa et al. 2015; van der Deen et al. 2006). Studies have also reported that MRP1 protects the heart against chronic doxorubicin-induced cardiotoxicity and protects intestinal epithelial cells against inflammation-induced apoptotic cell death (Blokzijl et al. 2008; Zhang et al. 2015). Thus, the noninvasive measurement of MRP1 activity in organs of interest would help to elucidate the pathogenesis and diagnoses of diseases.

6-Bromo-7-[^11^C]methylpurine ([^11^C]7m6BP) has been used to measure MRP1 activity in the brain and lungs of rodents (Krohn et al. 2019; Mairinger et al. 2020; Okamura et al. 2009b, 2013, 2020; Zoufal et al. 2019, 2020), and [^11^C]7m6BP is thus expected to be useful for assessment of the MRP1 activity in the tissues of human. Although a recent report has suggested a limited sensitivity of [^11^C]7m6BP for measuring MRP1 activity in the brain of a mouse species (Okamura et al. 2020), if the efflux process via MRP1 is the rate-limiting step in the human brain, it may be possible to assess the MRP1 activity in the brain. However, the low yield of the current synthetic method remains a problem, limiting the clinical use of [^11^C]7m6BP. In the conventional method using alkyl halides and bases, the alkylation of 6-chloropurine or 6-bromopurine results in a mixture of *N*7- and *N*9-alkylated purines, and the *N*9-isomers are predominantly produced (Galante et al. 2014; Hanna et al. 1994; Montgomery and Temple 1961; Tuncbilek et al. 2009; Zhang et al. 2012). Consistent with this production of isomers, the ^11^C-methylation of 6-bromopurine has resulted in a low radiochemical yield of [^11^C]7m6BP (Okamura et al. 2009b; Zoufal et al. 2019). In the present study, we investigated conditions for improving the [^11^C]7m6BP/[^11^C]9m6BP selectivity in this methylation reaction.

## Methods

### Chemicals

6-Bromopurine was purchased from Sigma-Aldrich/Merck (St. Louis, MO, USA). 6-Bromo-9-methylpurine and 6-bromo-7-methylpurine were synthesized as described previously (Okamura et al. 2009a, b). Chloroform (CHCl_3_, dehydrated) and super dehydrated solvents—acetone (ACT), acetonitrile (MeCN), ethyl acetate (AcOEt), *N,N*-dimethylformamide (DMF), tetrahydrofuran (THF), 1,4-dioxane (1,4-DO), diisopropyl ether (*i*Pr_2_O), toluene (Tol), and dichloromethane (DCM)—were purchased from FUJIFILM Wako Pure Chemical Corporation (Osaka, Japan). Methyl acetate (AcOMe, anhydrous) and 1,3-dioxane (1,3-DO) were purchased from Sigma-Aldrich/Merck. Methyl propionate (MP, special grade) was purchased from FUJIFILM Wako Pure Chemical Corporation. 2-Methyltetrahydrofuran (2-MeTHF, dehydrated) was purchased from KANTO CHEMICAL Co., INC (Tokyo, Japan). Aqueous 57% hydrogen iodide (HI) solution was purchased from FUJIFILM Wako Pure Chemical Corporation. Potassium Carbonate (K_2_CO_3_, guaranteed reagent) was purchased from FUJIFILM Wako Pure Chemical Corporation and was ground in a mortar into a fine powder, which was used for manual and automated syntheses. A 0.05–0.08 M solution of lithium aluminum hydride (LAH) in THF was prepared by diluting a 1.0 M solution of LAH in THF, which was purchased from Sigma-Aldrich/Merck.

### Production of [^11^C]carbon dioxide ([^11^C]CO_2_)

[^11^C]CO_2_ was produced by the ^14^N(p,α)^11^C nuclear reaction in an atmosphere of nitrogen gas containing 0.01% oxygen with 18 MeV protons using the CYPRIS HM-18 cyclotron (Sumitomo Heavy Industry, Tokyo, Japan).

### Production of [^11^C]CH_3_I

[^11^C]CH_3_I was prepared from [^11^C]CO_2_ as described previously, except for the volumes of the solutions of LAH in THF and 57% HI (Kikuchi et al. 2013). The volumes of the LAH in THF and 57% HI solutions used were both 100 µL. [^11^C]CH_3_I was transferred using a N_2_ gas stream with a flow rate of 50 mL/min and collected in MeCN (*ca*. 2 mL) in a glass vial.

### General conditions for ^11^C-methylation (manual synthesis)

The following solvents were investigated in this reaction: ACT; MeCN; DMF; AcOMe; AcOEt; MP; THF; 1,3-DO; 2-MeTHF; 1,4-DO; *i*Pr_2_O; CHCl_3_; Tol; and DCM. The [^11^C]CH_3_I solution (< 80 MBq, 10 μL) was added to a mixture of 6-bromopurine (5.1 mg, 25.6 µmol), K_2_CO_3_ (3.6 mg, 26.0 µmol), and each solvent (400 μL) in a 1.6-mL glass vial. The vial was sealed with a screw cap, and the temperature was then held at 100, 140, or 180 °C for 5 min. At the end of the reaction, the reaction mixture was cooled on ice, 200 μL of methanol was added, and the resulting mixture was then analyzed by both high-performance liquid chromatography (HPLC) and thin-layer chromatography (TLC).

### Radiochemical yields from [^11^C]CH_3_I

All radiochemical yields (RCYs) were determined by radio-HPLC analysis and radio-TLC of the crude product. HPLC was used for the separation of [^11^C]CH_3_I and the methylated products including the *N*7-isomer ([^11^C]7m6BP) and the *N*9-isomer (6-bromo-9-[^11^C]methylpurine; [^11^C]9m6BP). Because [^11^C]7m6BP and [^11^C]9m6BP could not be separated by the HPLC conditions used here, TLC was used for the separation of these compounds. RCYs based on [^11^C]CH_3_I were calculated as follows: RCY_HPLC_ multiplied by RCY_TLC_, where RCY_HPLC_ and RCY_TLC_ show the proportion of the ^11^C-methylated products determined by HPLC and that of [^11^C]7m6BP determined by TLC, respectively.

### HPLC analysis

The HPLC system consisted of a JASCO PU-2089 plus pump (JASCO Corporation, Tokyo, Japan), a multiwavelength detector (MD-2015 plus, JASCO Corporation), and a sensitive positron detector (Ohyo Koken Kogyo, Co., Ltd. Tokyo, Japan) for radioactivity detection. Data acquisition and interpretation were performed with ChromNAV (version 1.18.03, JASCO Corporation). The RCYs were calculated after the correction of the radiochromatograms for decay. A COSMOSIL 5C18-MS-II column (150 × 4.6 mm; Nacalai Tesque, Kyoto, Japan) and a 5C18-MS-II guard column (10 × 4.6 mm) were used for the RCY analyses with a mobile phase consisting of a mixture of water and MeCN. The peaks corresponding to the ^11^C-labeled compounds in the radiochromatogram were determined based on the UV absorptions of the corresponding nonradioactive compounds. The retention times of the ^11^C-methylated products ([^11^C]7m6BP and the *N*9-isomer) and [^11^C]CH_3_I (mobile phase: water/MeCN, 40/60; flow rate: 1 mL/min) were approximately 2 and 4 min, respectively.

### TLC analysis

TLC was conducted on glass-backed silica gel TLC plates (silica gel 60 F254; Merck Ltd., Tokyo, Japan), and unlabeled 7m6BP was pre-spotted on the TLC plates. The reaction solvents were used, except for DMF, and each reaction mixture (2 µL) was co-spotted with unlabeled 7m6BP. The TLC plates were developed with AcOEt/EtOH (9/1, v/v). When DMF was used as the reaction solvent, the reaction mixture was further diluted with methanol (four-fold dilution, in total six-fold dilution). One µL of the reaction mixture was co-spotted with unlabeled 7m6BP, and the TLC plates were developed with CHCl_3_/AcOEt (9/1, v/v). After drying, the plates were developed with AcOEt/EtOH (9/1, v/v) again. The air-dried TLC plates were covered with a 5 μm-thick film and placed in a cassette in contact with a phosphor imaging plate. Radioactivity on the TLC plate was quantified with an imaging plate reader (BAS-5000, FUJIFILM Corporation, Tokyo, Japan). The fraction of radioactivity on the TLC plates and a typical image of a TLC plate were shown in Additional file 1: Table S1 and Fig. S2, respectively.

### Automated synthesis of 6-bromo-7-[^11^C]methylpurine using [^11^C]methyl triflate

#### Production of [^11^C]methyl triflate

[^11^C]CH_3_I was produced as described above. [^11^C]Methyl triflate ([^11^C]CH_3_OTf) was prepared from [^11^C]CH_3_I as described previously (Jewett 1992). In brief, [^11^C]CH_3_OTf was produced by passing [^11^C]CH_3_I through a glass column containing silver triflate-impregnated graphitized carbon (200–300 mg) at 180 °C with a N_2_ flow of 50 mL/min.

#### ^11^C-methylation using a synthetic apparatus

Automated radiochemical synthesis was performed using a system built in-house (Additional file 1: Fig. S1). 6-Bromopurine (2 mg, 10.0 µmol), K_2_CO_3_ (6.4 mg, 26.0 µmol), and solvent (400 μL) were added into a 1.6-mL glass vial. The mixture was vortexted and sonicated at room temperature (20–23 °C) for approximately 20 s, to form a suspension. The suspension was drawn up into a 1-mL syringe with a 20G needle and then injected into a reaction vessel for the automated synthesis. When DMSO was used, the reaction conditions including the reaction temperature, the reaction time, and the solvent volume were according to a previous report (Zoufal et al. 2019).

[^11^C]CH_3_OTf was trapped in the precursor mixture at room temperature, and the reaction vessel was heated at 100 °C for 5 min. The reaction vessel was cooled to room temperature, 1 mL of AcOEt was added, and the radioactive mixture was transferred into a COSMOSIL 5SL-II column (10 ID × 250 mm; Nacalai Tesque). The column was eluted with AcOEt/EtOH (93:7, v/v) at a flow rate of 5.0 mL/min. The radioactive fraction corresponding to the desired product (retention time: approximately 10–12 min) was collected in a rotary evaporator flask containing 50 μL of 25% ascorbic acid injection, evaporated in vacuo, and dissolved in 5 mL of saline. The total synthesis time, after 10 min of proton bombardment with a beam current of 20 µA, was 29–32 min. The identification and radiochemical purity (RCP) of [^11^C]7m6BP obtained after the formulation were determined by HPLC using an authentic sample of 7m6BP. HPLC was performed on a COSMOSIL 5C18-AR-II column (4.6 I.D. × 250 mm; Nacalai Tesque) and a 5C18-AR-II guard column (10 × 4.6 mm) with a mobile phase of H_2_O/methanol (75:25, v/v) at a flow rate of 0.9 mL/min. The molar activity (*A*_m_) was determined by comparing the assayed radioactivity to the mass associated with the 7m6BP UV peak at 288 nm.

## Results

### Effect of the solvent on the RCY of [^11^C]7m6BP (manual synthesis)

When ACT, MeCN, or DMF was used for the ^11^C-methylation of 6-bromopurine, the reaction proceeded efficiently at 100 °C, and unreacted [^11^C]CH_3_I was not observed. However, the product ratio of [^11^C]7m6BP/[^11^C]9m6BP was low (Table 1, entries 1–3), and the RCYs of [^11^C]7m6BP were 24–26%. When *i*Pr_2_O, CHCl_3_, Tol, or DCM was used, a considerable amount of unreacted [^11^C]CH_3_I was observed, and the reaction scarcely proceeded even at high temperature (180 °C) (Table 1, entries 25–34). These results are likely to be because of the poor solubility of 6-bromopurine in these solvents. While 1,4-DO and 2-MeTHF improved the [^11^C]7m6BP/[^11^C]9m6BP selectivity to 2:1, the methylation did not proceed efficiently (Table 1, entries 19–24). The RCYs of [^11^C]7m6BP were thus low even at high temperature (180 °C), and were lower than those using ACT, MeCN, or DMF. The use of AcOMe, AcOEt, MP, or THF gave a [^11^C]7m6BP/[^11^C]9m6BP selectivity of > 1:1 (Table 1, entries 4–15). Although 32–76% of unreacted [^11^C]CH_3_I was observed at 100 °C, the reaction proceeded smoothly at higher temperatures (140 °C and 180 °C), and the amount of unreacted [^11^C]CH_3_I was reduced to 0.97–7.5%. Furthermore, 1,3-DO increased the selectivity compared with ACT, MeCN, and DMF; however, the ratio of [^11^C]7m6BP/[^11^C]9m6BP was still < 1:1 (Table 1, entries 16–18).

**Table 1:** Effect of the solvent and temperature on the RCY of [^11^C]7m6BP


Entry	Solvent	T (°C)	[^11^C]CH_3_I^b^	RCY (%)^a^		Ratio^c^
				[^11^C]7m6BP	[^11^C]9m6BP	
1	ACT	100	0.067	24	52	0.46
2	MeCN	100	0.036	25	51	0.49
3	DMF	100	0	26	65	0.40
4	AcOMe	100	49	24	21	1.1
5	AcOMe	140	7.5	41	39	1.1
6	AcOMe	180	0.036^d^	38	38	1.0
7	AcOEt	100	58	20	16	1.2
8	AcOEt	140	25	33	30	1.1
9	AcOEt	180	4.8	39	37	1.1
10	MP	100	76	12	9.4	1.3
11	MP	140	29	34	28	1.2
12	MP	180	6.8	42	35	1.2
13	THF	100	32	34	28	1.2
14	THF	140	6.6	45	38	1.2
15	THF	180	0.97	43	36	1.2
16	1,3-DO	100	7.4	35	43	0.82
17	1,3-DO	140	1.1	37	48	0.77
18	1,3-DO	180	0.14	30	38	0.78
19	2-MeTHF	100	92	4.1	2.2	1.9
20	2-MeTHF	140	80	12	6.0	2.0
21	2-MeTHF	180	61	19	10	1.9
22	1,4-DO	100	92	3.3	1.7	1.9
23	1,4-DO	140	81	9.3	4.7	2.0
24	1,4-DO	180	57	19	10	1.9
25	*i*Pr_2_O	100	98	Trace	Trace	–
26	*i*Pr_2_O	140	96	Trace	Trace	–
27	*i*Pr_2_O	180	94	Trace	Trace	–
28	CHCl_3_	100	95	Trace	Trace	–
29	CHCl_3_	140	94	Trace	Trace	–
30	CHCl_3_	180	94	Trace	Trace	–
31	Tol	100	96	Trace	Trace	–
32	Tol	140	96	Trace	Trace	–
33	Tol	180	94	Trace	Trace	–
34	DCM	100	98	Trace	Trace	–

[^11^C]7m6BP was manually synthesized using [^11^C]CH_3_I (*n* = 1)

^a^Determined by radio-HPLC and TLC analysis of the crude product

^b^Unreacted [^11^C]CH_3_I

^c^Ratio of [^11^C]7m6BP/[^11^C]9m6BP

^d^AcOMe in the vial sealed with a screw cap was evaporated under these conditions, and thus the calculation of the proportion of unreacted [^11^C]CH_3_I was not reliable

### Effect of the solvent on the isolated RCY of [^11^C]7m6BP (automated synthesis)

**Fig. 1:**
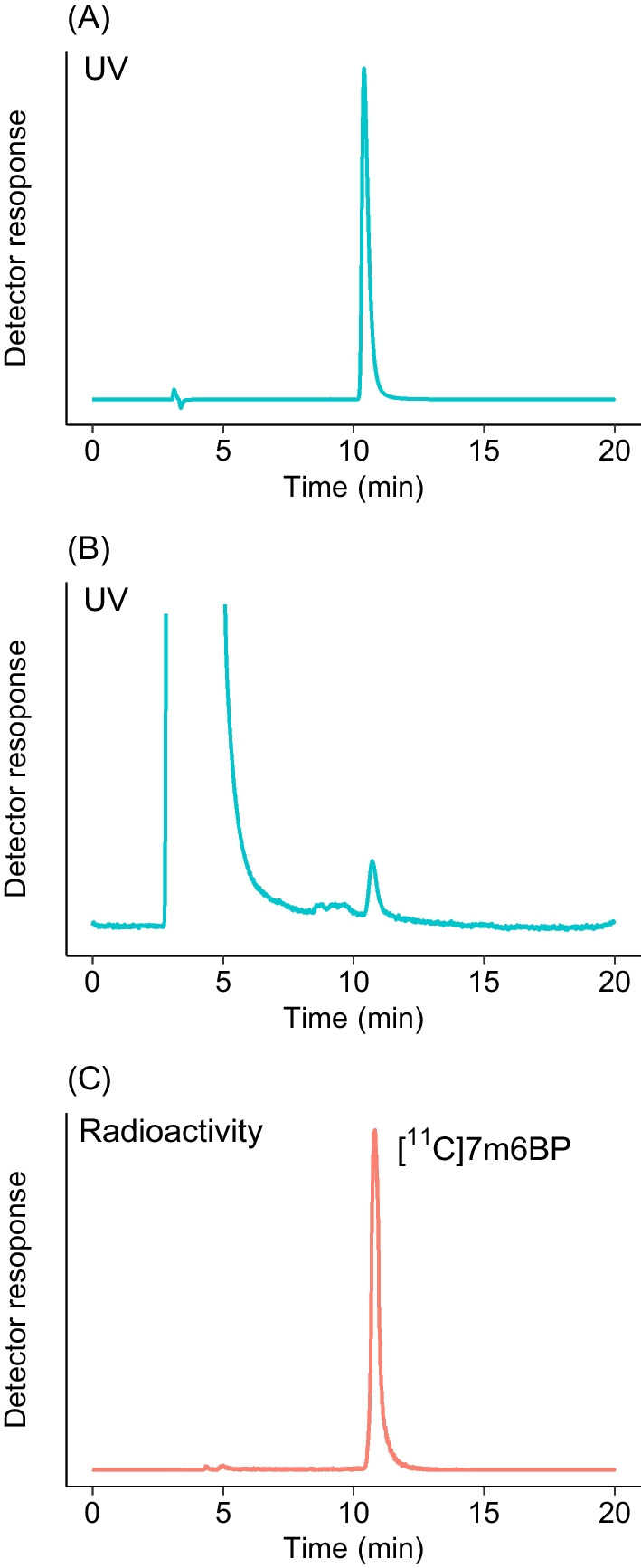
Representative analytical chromatograms of isolated [^11^C]7m6BP. UV-chromatogram of the authentic sample of 7m6BP (**A**). UV-chromatogram (**B**) and radiochromatogram (**C**) of isolated [^11^C]7m6BP immediately after synthesis. The UV absorbance was measured at 288 nm. The radiochromatogram was corrected for baseline noise and decay. The synthesis of [^11^C]7m6BP was performed using [^11^C]CH_3_OTf in THF at 100 °C

**Fig. 2:**
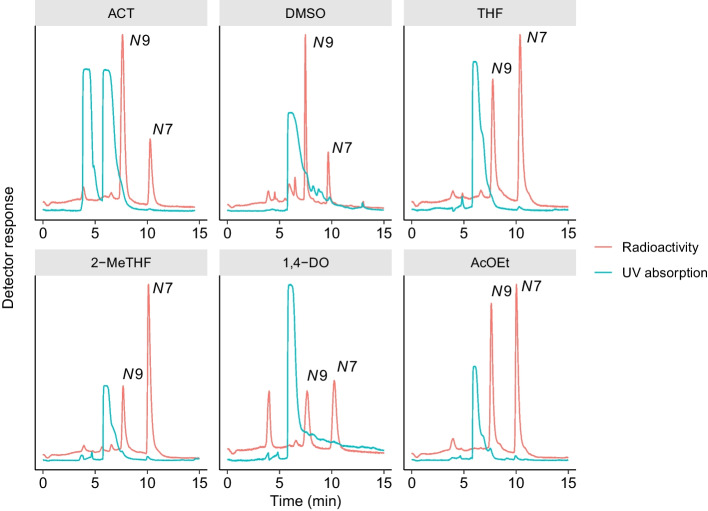
Typical chromatograms of semi-preparative HPLC for the reaction mixture. The peaks of *N*7 and *N*9 show [^11^C]7m6BP and [^11^C]9m6BP, respectively. ACT, DMSO, THF, 2-MeTHF, 1,4-DO, and AcOEt were used as reaction solvents

From the results of the manual synthesis, the conditions in which the reaction was performed in THF at 140 °C (Table 1, entry 14) appeared to be appropriate for the production of [^11^C]7m6BP. However, such reaction conditions that generated a high pressure were not suitable for [^11^C]7m6BP production using the apparatus for the automated synthesis. In the automated synthesis, [^11^C]7m6BP was therefore synthesized using [^11^C]CH_3_OTf and THF at a lower temperature (100 °C), and the tracer was successfully obtained under these conditions (Table 2 and Fig. 1). The reactivity of 6-bromopurine with [^11^C]CH_3_I at 100 °C in AcOEt was much lower than in THF, and 58% of [^11^C]CH_3_I was unreacted (Table 1, entry 7). However, the reaction with [^11^C]CH_3_OTf in the automated synthesis proceeded efficiently, and the selectivity remained almost unchanged (Table 2 and Fig. 2). The isolated RCY of [^11^C]7m6BP in AcOEt was comparable to that in THF (Table 2). The use of 2-MeTHF and [^11^C]CH_3_OTf improved the low reactivity with a similar [^11^C]7m6BP/[^11^C]9m6BP selectivity (Table 2 and Fig. 2). By contrast, the reaction using 1,4-DO and [^11^C]CH_3_OTf was unsuccessful, and a peak of an unknown ^11^C-labeled by-product of comparable intensity to [^11^C]7m6BP and [^11^C]9m6BP was observed (Fig. 2), which was probably a decomposed product derived from unreacted [^11^C]CH_3_OTf. When ACT or DMSO was used as the solvent, the isolated RCYs of [^11^C]7m6BP were low (Table 2). Taken together, these results indicated that the use of THF, 2-MeTHF, or AcOEt increased the isolated RCY of [^11^C]7m6BP. Starting from 28 to 34 GBq [^11^C]CO_2_, [^11^C]7m6BP was produced with 2.3–2.6 GBq using THF, 2.7–3.3 GBq using AcOEt, and 2.8–3.9 GBq using 2-MeTHF at approximately 30 min after the end of bombardment (EOB). The isolated RCYs (decay corrected) for THF, 2-MeTHF, and AcOEt were 24–28%, 29–35%, and 22–31% (*n* = 3), respectively. The radiochemical purity of [^11^C]7m6BP was higher than 95% up to 60 min after being formulated (Table 2), indicating the radiochemical stability of the compound for the duration of at least one positron emission tomography scan.

**Table 2:** Yield, purity, and molar activity of [^11^C]7m6BP after purification and formulation


Solvent	Activity (GBq)^a^	Yield (GBq)^b^	Time (min)^c^	Yield (%)^d^	RCP (%)^e^	A_m_ (GBq/µmol)^f^	Ratio^g^
ACT	28	1.1	31	11	99	130	0.41
ACT	28	0.95	30	9.2	99	79	0.40
ACT	29	0.65	32	6.6	99	170	0.40
DMSO	29	0.50	29	4.6	97	100	0.42
DMSO	29	0.82	29	7.7	92	140	0.52
DMSO	29	0.87	31	8.7	87	130	0.62
THF	28	2.3	32	24	95	170	1.3
THF	28	2.6	32	28	95	140	1.5
THF	29	2.6	32	26	96	180	1.2
2-MeTHF	29	2.9	31	29	96	200	2.0
2-MeTHF	33	3.9	31	35	95	150	2.2
2-MeTHF	28	2.8	31	29	97	98	1.7
1,4-DO	30	1.1	31	11	98	140	1.3
1,4-DO	30	1.2	30	11	96	190	1.3
1,4-DO	29	1.1	31	11	95	160	1.3
AcOEt	34	2.7	30	22	98	160	1.2
AcOEt	31	3.3	32	31	98	140	1.2
AcOEt	29	3.0	31	30	97	110	1.3

Automated synthesis of [^11^C]7m6BP using [^11^C]CH_3_OTf (*n* = 3)

^a^Initial activity of [^11^C]CO_2_ at EOB

^b^Isolated yield at end of synthesis (EOS)

^c^Total synthesis time from EOB to EOS

^d^Isolated yield (decay corrected to the EOB)

^e^Radiochemical purity at 60 min after synthesis

^f^Molar activity at EOS

^g^Ratio of the peak area for [^11^C]7m6BP to [^11^C]9m6BP calculated from each HPLC chromatogram

## Discussion

In the present study, we investigated conditions for improving the [^11^C]7m6BP/[^11^C]9m6BP of selectivity in the methylation reaction in the synthesis of [^11^C]7m6BP. The methylation of the precursor with [^11^C]CH_3_I in ACT, MeCN, or DMF provided primarily [^11^C]9m6BP (Table 1, entries 1–3), and the reaction in *i*Pr_2_O, CHCl_3_, Tol, or DCM scarcely proceeded (entries 25–34). Although 1,4-DO and 2-MeTHF improved the [^11^C]7m6BP/[^11^C]9m6BP selectivity to 2:1, the methylation did not proceed efficiently (entries 19–24). The use of less polar solvents—AcOMe, AcOEt, MP, THF, and 1,3-DO—appeared promising for increasing the RCY of [^11^C]7m6BP at 140 °C or 180 °C. From the results of the manual synthesis, we selected the conditions using THF at 140 °C (entry 14), which might increase the isolated RCY of [^11^C]7m6BP, compared with previous methods for the synthesis of [^11^C]7m6BP (Okamura et al. 2009b; Zoufal et al. 2019). In a preliminary examination, however, a considerable loss of radioactivity in the reaction vial during the automated synthesis was observed. This loss resulted from the passing of [^11^C]CH_3_I through a solenoid valve before the precursor was methylated, probably because of the high pressure caused by heating THF (boiling point: 66 °C) at 140 °C. The temperature parameter from the manual synthesis could thus not be translated directly to the automated synthesis. While a lower temperature was required for the automated synthesis, a higher temperature was needed for the efficient methylation of 6-bromopurine (Table 1). The methylation of 6-bromopurine with [^11^C]CH_3_I in THF at 100 °C proceeded moderately (Table 1, entry 13), but the methylation at 80 °C scarcely proceeded (data not shown). The [^11^C]7m6BP/[^11^C]9m6BP selectivity was almost constant from 100 to 180 °C (Table 1, entries 13–15). In the automated synthesis, the temperature was thus changed from 140 to 100 °C, and [^11^C]CH_3_OTf replaced [^11^C]CH_3_I as the methylating agent to compensate for the reactivity loss. [^11^C]7m6BP was successfully obtained under these conditions, and the selectivity of [^11^C]7m6BP/[^11^C]9m6BP in THF in the automated synthesis (Table 2) was consistent with that in the manual synthesis (Table 1, entry 13).

In addition to THF, the ^11^C-methylation at 100 °C proceeded efficiently in 2-MeTHF and AcOEt, while maintaining the improved selectivity (Table 2 and Fig. 2). By contrast, when ACT was used as the solvent, the isolated RCYs of [^11^C]7m6BP were 6.6–11%, which were a little higher than those in a previous study using ACT and [^11^C]CH_3_I (4–9%) (Okamura et al. 2009b). This increase may be because there were differences in the reactivity between [^11^C]CH_3_OTf and [^11^C]CH_3_I but the selectivity was not changed. The [^11^C]7m6BP/[^11^C]9m6BP selectivity was similar in the automated synthesis to that in the manual synthesis (Tables 1 and 2). When DMSO was used as the solvent, the radiochemical purity of [^11^C]7m6BP (two out of three runs) did not achieve 95%, because of contamination with the *N*9-isomer. Aside from the radiochemical purity, the isolated RCY of [^11^C]7m6BP in DMSO was still low, which was consistent with a previous study (Zoufal et al. 2019).

Chen et al. have reported that treating 6-bromopurine with an alkylmagnesium reagent, which was then reacted with CH_3_I at 25 °C for 20 h, gave the *N*7-isomer (Chen et al. 2016). If 6-bromopurine can be reacted with [^11^C]CH_3_OTf or [^11^C]CH_3_I at a lower temperature (room temperature) and for a longer time, [^11^C]7m6BP might be formed more selectively. However, such a long reaction time is impractical for the radiosynthesis of [^11^C]7m6BP. Although our conditions did not provide only [^11^C]7m6BP, the reaction can potentially produce sufficient [^11^C]7m6BP for clinical use (Table 2).

The present study showed that the solvents, THF, 2-MeTFH, and AcOEt, increased the [^11^C]7m6BP/[^11^C]9m6BP selectivity in the methylation of 6-bromopurine, compared with the traditionally used solvents, ACT, MeCN, DMF, and DMSO. The mechanism of the reaction remains unknown; however, the solvent and temperature might affect the relative populations of the tautomers (6-bromo-7*H*-purine and 6-bromo-9*H*-purine). To the best of our knowledge, no reports are available on the determination of tautomerism in 6-bromopurine in any solvent. For 6-chloropurine, the population of the 7*H* tautomer has been reported to be 8–22% at 303 K (30 °C) in DMSO (Seckarova et al. 2004), which probably led to the low yield of 6-chloro-7-alkylpurine (Montgomery and Temple 1961). The population of the 7*H* tautomer of 6-bromopurine is thus assumed to be comparable to, or higher than, that of the 9*H* tautomer in the less polar solvents, such as THF, 2-MeTFH, and AcOEt, whereas the 9*H* tautomer may be the dominant tautomeric form in more polar solvents.

## Conclusions

The use of 2-MeTHF, THF, and AcOEt improved the [^11^C]7m6BP/[^11^C]9m6BP selectivity in the methylation reaction. The reaction conditions for providing [^11^C]7m6BP with sufficient radioactivity for clinical use are the use of 2-MeTHF (THF or AcOEt), [^11^C]CH_3_OTf as a methylating agent, a reaction temperature of 100 °C, and a reaction time of 5 min.

### Supplementary Information

**Additional file 1**. **Table S1**: Fraction of [^11^C]7m6BP determined by TLC; **Figure S1**: Schematic diagram of the automated system used for [^11^C]7m6BP synthesis; **Figure S2**: Typical image of a developed TLC plate spotted with a reaction mixture.

## Data Availability

The datasets used and/or analyzed during the current study are available from the corresponding author on reasonable request.

## Abbreviations

1,3-DO: 1,3-Dioxane
1,4-DO: 1,4-Dioxane
2-MeTHF: 2-Methyltetrahydrofuran
[^11^C]7m6BP: 6-Bromo-7-[^11^C]methylpurine
[^11^C]9m6BP: 6-Bromo-9-[^11^C]methylpurine
ACT: Acetone
AcOEt: Ethyl acetate
AcOMe: Methyl acetate
DCM: Dichloromethane
DMF: *N,N*-Dimethylformamide
EOB: End of bombardment
EOS: End of synthesis
HI: Hydrogen iodide
HPLC: High-performance liquid chromatography
*i*Pr_2_O: Diisopropyl ether
MeCN: Acetonitrile
MP: Methyl propionate
MRP1: Multidrug resistance-associated protein 1
LAH: Lithium aluminum hydride
RCP: Radiochemical purity
RCY: Radiochemical yield
THF: Tetrahydrofuran
TLC: Thin-layer chromatography
Tol: Toluene
UV: Ultraviolet
