# Supplementary material for: Improved synthesis of 6-bromo-7-[11C]methylpurine for clinical use
Source: EJNMMI Radiopharm Chem. 2024 Feb 9;9:10. doi: 10.1186/s41181-024-00240-8 (PMC10857989; doi:10.1186/s41181-024-00240-8)
Supplement: Supplementary file 1 — Additional file 1. Table S1: Fraction of [11C]7m6BP determined by TLC; Figure S1: Schematic diagram of the automated system used for [11C]7m6BP synthesis; Figure S2: Typical image of a developed TLC plate spotted with a reaction mixture. [file 41181_2024_240_MOESM1_ESM.docx]

Supplementary material

**Improved synthesis of 6-bromo-7-[^11^C]methylpurine for clinical use**

Toshimitsu Okamura^a,^*, Tatsuya Kikuchi^a^, Masanao Ogawa^a,b^, and Ming-Rong Zhang^a^

^a^Department of Advanced Nuclear Medicine Sciences, Institute for Quantum Medical Science, National Institutes for Quantum Science and Technology, 4-9-1 Anagawa, Inage-ku, Chiba 263-8555, Japan.

^b^SHI Accelerator Service, Ltd., 7-1-1 Nishigotanda, Shinagawa-ku, Tokyo 141-0031, Japan

*Correspondence: Toshimitsu Okamura; E-mail: okamura.toshimitsu@qst.go.jp

**Table of Contents**

1 Table S1. Fraction of [^11^C]7m6BP determined by TLC S1

2 Figure S1. Schematic diagram of the automated system used for [^11^C]7m6BP synthesis S2

3 Figure S2. Typical image of a developed TLC plate spotted with a reaction mixture S3

4 Reference S4

**Table S1**. Fraction of [^11^C]7m6BP determined by TLC*^a^*.

| Entry | Solvent | T (°C) | Fraction (%)*^b^* | | Ratio*^c^* |
| --- | --- | --- | --- | --- | --- |
|  |  |  | [^11^C]7m6BP | [^11^C]9m6BP |  |
| 1 | ACT | 100 | 24 | 53 | 0.45 |
| 2 | MeCN | 100 | 25 | 52 | 0.48 |
| 3 | DMF | 100 | 26 | 65 | 0.40 |
| 4 | AcOMe | 100 | 48 | 43 | 1.1 |
| 5 | AcOMe | 140 | 46 | 43 | 1.1 |
| 6 | AcOMe | 180 | 39 | 38 | 1.0 |
| 7 | AcOEt | 100 | 50 | 40 | 1.2 |
| 8 | AcOEt | 140 | 46 | 41 | 1.1 |
| 9 | AcOEt | 180 | 42 | 40 | 1.0 |
| 10 | MP | 100 | 51 | 39 | 1.3 |
| 11 | MP | 140 | 49 | 40 | 1.2 |
| 12 | MP | 180 | 46 | 39 | 1.2 |
| 13 | THF | 100 | 51 | 42 | 1.2 |
| 14 | THF | 140 | 49 | 41 | 1.2 |
| 15 | THF | 180 | 44 | 37 | 1.2 |
| 16 | 1,3-DO | 100 | 39 | 47 | 0.83 |
| 17 | 1,3-DO | 140 | 38 | 49 | 0.78 |
| 18 | 1,3-DO | 180 | 30 | 39 | 0.77 |
| 19 | 2-MeTHF | 100 | 54 | 29 | 1.9 |
| 20 | 2-MeTHF | 140 | 59 | 30 | 2.0 |
| 21 | 2-MeTHF | 180 | 53 | 28 | 1.9 |
| 22 | 1,4-DO | 100 | 44 | 23 | 1.9 |
| 23 | 1,4-DO | 140 | 52 | 26 | 2.0 |
| 24 | 1,4-DO | 180 | 49 | 26 | 1.9 |

*^a^* [^11^C]7m6BP was manually synthesized using [^11^C]CH_3_I (*n* = 1).

*^b^* fraction of radioactivity on the TLC-plates.

*^c^* ratio of [^11^C]7m6BP/[^11^C]9m6BP.

**Figures**

**Figure S1**. Schematic diagram of the automated system used for [^11^C]7m6BP synthesis.

Automated radiochemical synthesis was performed using a system built in-house. [^11^C]CH_3_I and [^11^C]7m6BP was prepared in the Vessel 1 and the Vessel 2, respectively. The schematic diagram was almost the same as previously reported (Kikuchi et al., 2022).

**Figure S2**. Typical image of a developed TLC plate spotted with a reaction mixture.

Lane 1 shows a reaction mixture. Lanes 2 and 3 show the spot corresponding to authentic unlabeled 7m6BP and 9m6BP, respectively.

**Reference**

Kikuchi T, Ogawa M, Okamura T, Gee AD, Zhang MR. Rapid ‘on-column’ preparation of hydrogen [^11^C]cyanide from [^11^C]methyl iodide via [^11^C]formaldehyde. Chem Sci. 2022;13(12):3556–62.
